# Non-Invasive Hemodynamic Monitoring in Transcatheter Aortic Valve Implantation

**DOI:** 10.3390/jcm14113794

**Published:** 2025-05-28

**Authors:** Thorald Stolte, Janarthanan Sathananthan, Jakob Johannes Reichl, Jasper Boeddinghaus, Max Wagener, Christian Schöpflin, Christoph Kaiser, Gregor Leibundgut, Felix Mahfoud, David Wood, John G. Webb, Thomas Nestelberger

**Affiliations:** 1 Department of Cardiology and Cardiovascular Research Institute Basel (CRIB), University Hospital Basel, University of Basel, 4031 Basel, Switzerland; thorald.stolte@usb.ch (T.S.); jakobjohannes.reichl@usb.ch (J.J.R.); jasper.boeddinghaus@usb.ch (J.B.); max.wagener@usb.ch (M.W.); christoph.kaiser@usb.ch (C.K.); gregor.leibundgut@usb.ch (G.L.); felix.mahfoud@usb.ch (F.M.); 2 Centre for Heart Valve Innovation, St. Paul’s Hospital, University of British Columbia, Vancouver, BC V6T 1Z4, Canada; jsathananthan@providencehealth.bc.ca (J.S.); david.wood@vch.ca (D.W.);; 3 Department of Cardiac Surgery, University Hospital Basel, University of Basel, 4031 Basel, Switzerland; hanschristian.schoepflin@usb.ch

**Keywords:** transcatheter aortic valve replacement, non-invasive monitoring, aortic valve gradients, cardiac output, cardiac index, stroke volume, blood pressure

## Abstract

**Background/Objectives:** Aortic valve stenosis (AS) is a prevalent cardiovascular condition among elderly patients frequently treated with Transcatheter Aortic Valve Implantation (TAVI). Traditional hemodynamic monitoring during TAVI relies on invasive methods. The ClearSight^®^ Finger Cuff system offers a non-invasive alternative for continuous hemodynamic monitoring. To compare the reliability and feasibility of non-invasive hemodynamic monitoring with traditional invasive hemodynamic monitoring during TAVI procedures. **Methods:** In this prospective observational study, patients undergoing elective TAVI were recruited from two tertiary hospitals between March and August 2023. Invasive hemodynamic measurements were obtained using arterial and pigtail catheters, with a subset undergoing right heart catheterization. Non-invasive measurements were captured using the ClearSight^®^ system. Data on baseline characteristics, procedural details, and 30-day follow-up outcomes were collected. **Results:** The study cohort comprised 50 patients (median age 82 years (IQR 78.0, 85.8), 50% female). Non-invasive measurements of cardiac output (CO), cardiac index (CI), and stroke volume (SV) were consistently lower than invasive measurements (CO: 4.1 vs. 4.8 L/min, *p* = 0.03; CI: 2.2 vs. 2.7 L/min/m^2^, *p* = 0.01, SV: 66 vs. 77 mL, *p* = 0.25). Non-invasive blood pressure readings were lower than invasive radial and aortic measurements before and after TAVI. Correlation of non- and invasive measurements was low but similar before and after TAVI (Mean percentage error of 52%). **Conclusions:** The ClearSight^®^ system provided lower absolute values for all evaluated hemodynamic parameters as well as low correlation compared to traditional methods pre- as well as post-interventional.

## 1. Introduction

Aortic valve stenosis (AS) is one of the most common valvular diseases and is the third most common cardiovascular disease in developed countries [1,2]. Transcatheter Aortic Valve Implantation (TAVI) has emerged as an efficient and safe minimally invasive therapeutic alternative to surgical aortic valve replacement (SAVR) [3,4]. Due to clinical advancements in the peri-procedural management of TAVI, current strategies predominantly rely on invasive techniques. Right heart catheterization to provide additional hemodynamic information, such as cardiac output, cardiac index (CI), or stroke volume (SV) for procedural guidance, is rarely performed during TAVI given its invasive nature. Although it enables precise hemodynamic measurements, right heart catheterization results in prolonged preparation time and is not without risks, including procedural complications, such as vascular trauma, thrombus formation, hemorrhage, and infection, especially in an already susceptible patient cohort, underscoring the imperative for non-invasive monitoring alternatives [4,5,6,7].

The ClearSight^®^ Finger Cuff system represents a non-invasive modality for the continuous assessment of several hemodynamic parameters using an inflatable cuff [8,9,10]. Empirical comparisons have demonstrated significant concordance between hemodynamic data acquired via the ClearSight^®^ system and those obtained through established invasive methodologies, indicating its potential to offer a reliable and continuous non-invasive alternative to traditional invasive monitoring approaches in patients undergoing TAVI without the risk of infection and thrombus formation [11,12,13,14,15,16].

This study aims to directly compare the reliability and feasibility of a novel non-invasive hemodynamic monitoring tool with invasive measurements for monitoring during TAVI procedures.

## 2. Materials and Methods

### 2.1. Study Design and Patient Cohort

In this prospective observational study, patients with severe aortic stenosis undergoing elective TAVI were recruited at the University Hospital Basel in Switzerland and at the Vancouver General Hospital in Canada. Unstable patients were excluded if they did not undergo elective TAVI or if they had severe tricuspid or mitral regurgitation or severe mitral valve stenosis. Informed consent was obtained from all patients. The TAVI procedure itself, including choice of access, device, and sedation management was at the discretion of the treating physician. Follow-up was performed 30 days after the procedure.

### 2.2. Invasive Hemodynamic Measurements

All patients received arterial measurements using a left radial artery catheter and aortal measurements using a pigtail catheter inserted from the contralateral femoral side or right radial side into the ascending aorta. In a subset of patients, a right heart catheterization (RHC) was also performed to determine hemodynamic parameters, such as cardiac output (CO) and cardiac index (CI), using Fick’s principle [17]. These measurements were taken a few minutes before and after valve deployment.

### 2.3. Non-Invasive Hemodynamic Measurements

**Figure 1:**
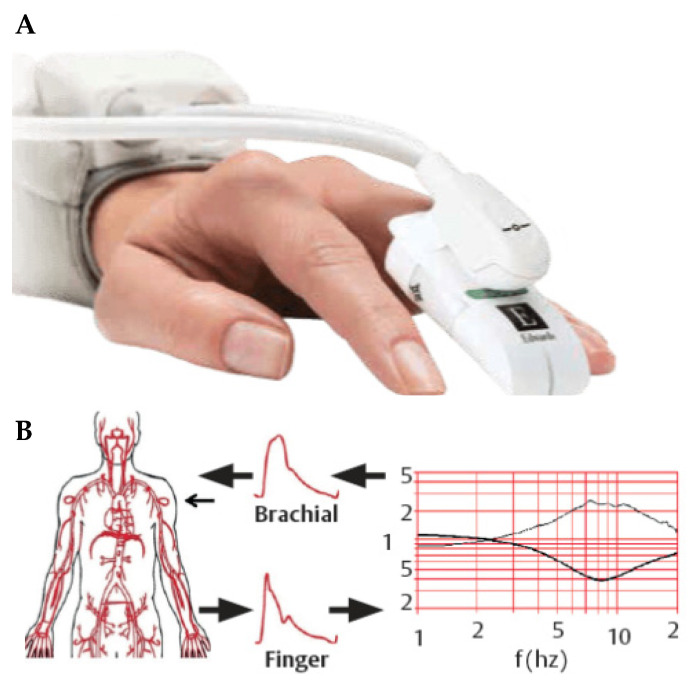
(**A**) ClearSight^®^ finger cuff and (**B**) schematic of the reconstruction of brachial arterial pressure waveform from finger arterial pressure waveform [18].

The ClearSight^®^ system (Edwards Lifesciences, Irvine, CA, USA) works non-invasively by utilizing an inflatable cuff which adjusts automatically to measure the arterial blood pressure waveform over the full pulse, based on the Peňáz principle (Figure 1). In this cuff, there is an infrared photodiode and light detector that measures the diameter of the finger artery [8]. It is the absorption of the infrared light through the finger that is used to measure the arterial volume [16]. Cuff pressure is constantly adjusted during the cardiac cycle to keep the volume in the finger constant and these adjustments over time are used to create the blood pressure waveform and enable analysis of the pulse wave [15]. Different pathophysiological changes affect the arterial compliance and resistance, but the ClearSight^®^ system can adjust to these changes. ClearSight^®^ is able to provide information regarding blood pressure, heart rate, SV, CO, cardiac index (CI), indexed stroke volume (SVI), stroke volume variation (SVV), systemic vascular resistance (SVR) and SVR index [8].

The ClearSight^®^ cuff was applied to the patient’s finger according to the instructions for use at the beginning of the TAVI procedure. Based on the standard setup of the operation room, the left index finger was used for all patients. The cuff was then connected to the monitor. The monitor unit recorded the measurements continuously during the whole procedure and saved all measurements. After the procedure, all measurements were assigned to the study-specific study ID and transferred to the database.

### 2.4. Data Collection and Follow-Up

Baseline characteristic, imaging data from transthoracic echocardiography before and after the procedure, laboratory results, procedural data, outcome data as well as follow-up information within 30 days after the procedure have been collected. Adverse events were systematically collected during the 30 days of follow-up and adjudicated by a dedicated clinical event committee based on detailed documentation of reported endpoints using the VARC-3 definitions.

### 2.5. Study Objectives

The first aim of this study was to test the correlation of non-invasive measurements (ClearSight^®^) compared with invasive measurements (radial and aortal measurements). Systolic and diastolic blood pressure, heart rate, CO, CI, and SV measured with ClearSight^®^ and RHC were compared at a certain timepoint a few minutes before and after valve implantation.

The second aim was to show trends of hemodynamic parameters (systolic and diastolic BP, CO, CI, HR, and SV) during the whole procedure and especially during rapid pacing and valve implantation.

### 2.6. Statistical Analysis

Categorical variables are shown as count (percentage), and continuous variables as median (interquartile range [IQR]). Comparisons between continuous variables were performed using the Mann–Whitney U test. Categorical variables were compared using Pearson’s Chi-squared test or Fisher’s exact test, as appropriate. Absolute and relative differences in patients’ hemodynamic parameters measured invasively and non-invasively immediately during the TAVI procedure were calculated and illustrated with Bland–Altman plots. The absolute bias was defined as the differences between invasive and non-invasive measurements and the precision as standard deviation (SD). The lower and upper limits of agreement (LoA) were defined as 1.96 × SD. Coefficients of error (CE) were calculated by dividing the SD of the differences by the mean of the measurements, while the percentages of error (PE) were defined as 2 × CE × 100. Acceptable levels of correlation were defined as an absolute bias of 20% and a PE of 30%, as previously used by Di Cori et al. [19]. A *p* value < 0.05 was considered statistically significant. All statistical analyses were performed using R 4.2.3 (R Foundation for Statistical Computing, Vienna, Austria).

## 3. Results

### 3.1. Baseline Patient and Procedural Characteristics

Patients were recruited from March to August 2023. Median age was 82.0 [78, 86] years old and 50% were female. Median Euro SCORE II was 1.90 [1.40, 4.30], while the STS calculated risk of mortality was 2.40 [1.65, 4.02].

Median left ventricular ejection fraction (LVEF) was 60% and the majority of patients (94%) had a NYHA-Score of II or more. No patients had undergone previous aortic valve surgery or TAVI (Table 1). 36% of patients had coronary artery disease and 14% underwent revascularization. Atrial fibrillation or flutter was present in 18% of patients.

All patients underwent femoral route access. A total of 32 patients (64%) received a SAPIEN S3 or SAPIEN S3 Ultra valve, 10 (20%) an Evolut pro+, and 8 (16%) a Acurate NEO 2 device. The most common prosthesis sizes were 23 and 26 mm (8 and 11 patients).

### 3.2. Continuous Non-Invasive Measurements

**Figure 2:**
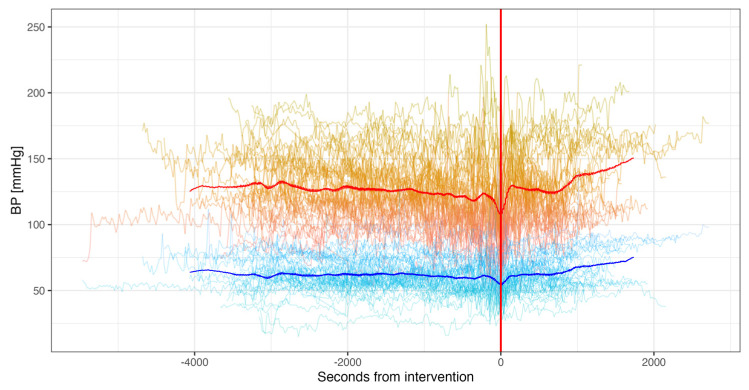
Mean continuous non-invasive (ClearSight^®^) blood pressure measurements from pre- to post-interventional. (Valve deployment marked with red line, systolic blood pressure in red, diastolic in blue).

**Figure 3:**
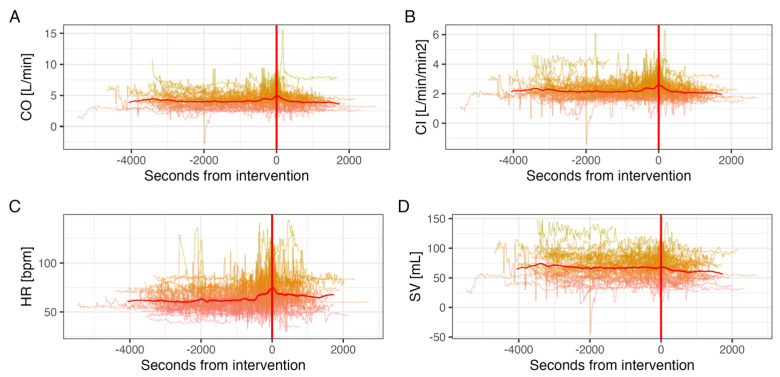
Mean continuous non-invasive (ClearSight^®^) (**A**) cardiac output, (**B**) cardiac index, (**C**) heart rate, and (**D**) stroke volume measurements from pre-to post interventional. (Valve deployment marked with red line).

Continuous non-invasive measurements of systolic and diastolic BP, CO, CI, HR and SV were collected for all 50 participants. Patients had a mean BP of 125/61 ± 26/13 mmHg before valve implantation. After implantation, patients had a mean BP of 130/64 ± 28/15 mmHg (Figure 2). CI and CO remained similar between the pre- and post-implantation period (Figure 3A,B). HR was significantly higher pre-implantation than post-implantation (65 vs. 60 bpm, *p* < 0.001) (Figure 3C). SV decreased continuously from pre- to post-implantation (67 vs. 60 mL, *p* < 0.001) (Figure 3D).

### 3.3. Agreement Between Non-Invasive, Aortal, and Radial Measurements

**Figure 4:**
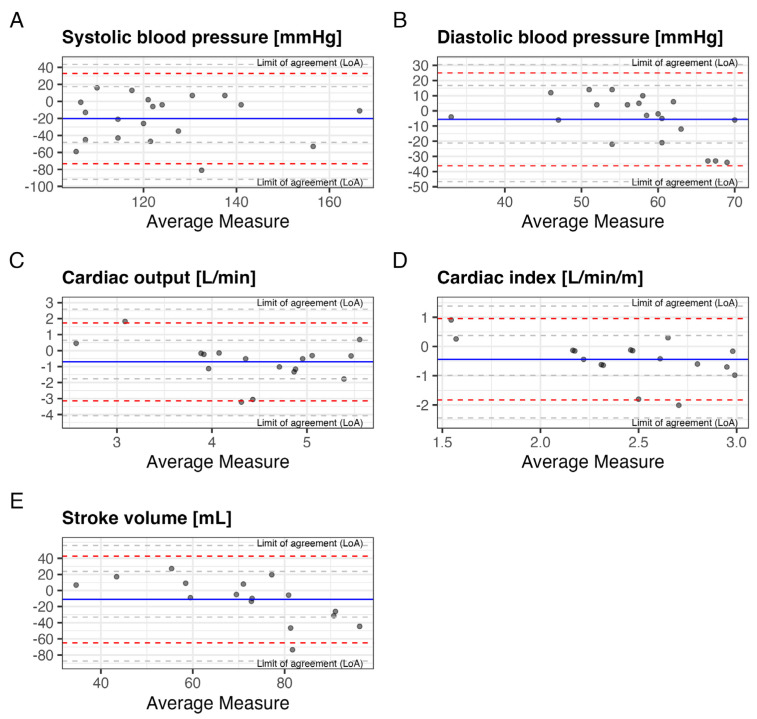
Bland–Altman plots comparing aortal to non-invasive (ClearSight^®^) measurements of (**A**) Systolic, (**B**) diastolic blood pressure, (**C**) cardiac output, (**D**) cardiac index and (**E**) stroke volume.

**Figure 5:**
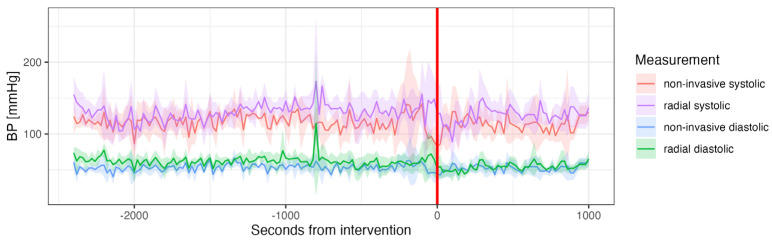
Comparison of mean measurements of radial and non-invasive (ClearSight^®^) blood pressure measurements and standard deviation over time. (Valve deployment marked with red line).

**Figure 6:**
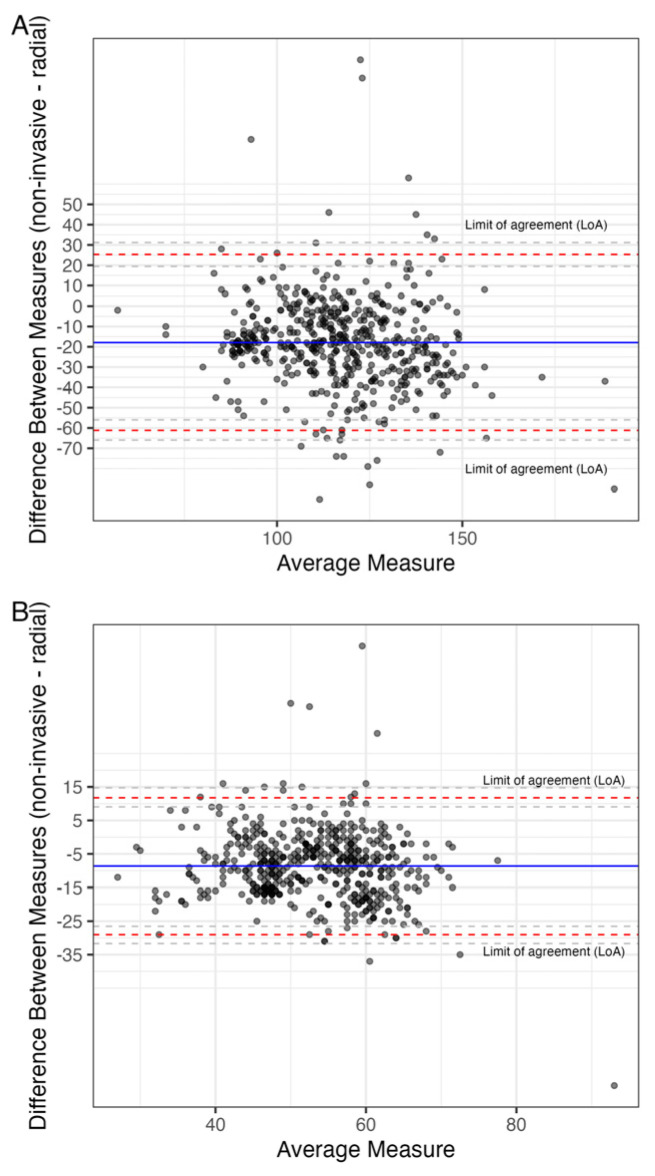
Bland–Altman plots comparing radial to non-invasive (ClearSight^®^) measurements of (**A**) systolic and (**B**) diastolic blood pressure.

A comparison between non-invasive and radial measurements was performed in 25 patients. Invasive measurements of systolic BP, CO and CI were significantly higher than non-invasive ones (132 vs. 113 mmHg, *p* = 0.004; 4.8 vs. 4.1 L/min, *p* = 0.03, and 2.7 vs. 2.2 L/min/m^2^, *p* = 0.01). Invasive measurements of diastolic BP and SV were similar to non-invasive ones (59.5 vs. 57 mmHg, *p* = 0.24, and 77 vs. 66 mL, *p* = 0.25). (Figure 4A–E) Comparisons of continuous radial to non-invasive measurements of systolic and diastolic blood pressure revealed consistently higher values for radial measurements in both systolic, as well as diastolic measurements (127/60 vs. 113/52 mmHg, *p* < 0.001 for both), with no difference from before to after valve deployment. There was no significant difference between aortal and radial BP measurements (Figure 5 and Figure 6A,B). None of the observed values showed correlation within the pre-defined acceptable levels of absolute bias (20%) and PE (30%). (Mean PE 52%) A summary table of all results from performed Bland–Altman analyses is given in Table 2.

### 3.4. Outcomes

Device success was achieved in all patients. Follow-up information at 30 days was available in all patients. Patients were discharged on average after 3 days [1, 5]. Mean gradient at discharge was 10 [8, 15] mmHg. During the 30-day follow-up, no patient died or suffered any major bleeding, myocardial infarction, stroke, or other adverse events.

## 4. Discussion

In this study, we aimed to investigate the utility of non-invasive compared to invasive hemodynamic measurements among 50 patients undergoing TAVI at two large, tertiary university hospitals. We report five major findings:

First, median systolic and diastolic radial, as well as aortic BP measurements were consistently and significantly higher compared to non-invasive measurements (127/60 and 132/60 vs. 113/52 mmHg, *p* < 0.001; absolute SD: 22.07/10.41 mmHg and 27.15/15.61 mmHg, PE: 38/39% and 44/55%). Di Cori et al. found similar results in patients both with and without atrial fibrillation [19], while Yahagi and Sasaki, and Vos et al. reported comparable percentage errors for systolic and diastolic BP (24.9% and 31.9%) [15,20]. Radial (systolic) BP tends to be higher than central aortal BP due to pulse pressure amplification, which occurs when the arterial pulse wave travels from the central aorta to the peripheral arteries. This results in higher systolic and lower diastolic pressures at peripheral sites like the radial artery. This consistent finding may therefore be attributed to the anatomical and physiological differences in pressure waveforms between the radial artery and central circulatory measurements. Recent publications, however, have shown a much narrower difference between systolic aortic and radial BP measurements, and no differences in mean and diastolic BP, highlighting the need for further research on the topic [21]. Furthermore, differences in arterial stiffness could contribute to discrepancies in measurements [22].

Second, CO, CI and SV measurements were slightly higher when obtained through invasive methods compared to non-invasive measurements (mean bias of −62% for non-invasive measurements and mean PE of 51%). Prior research has demonstrated ClearSight^®^ to be both accurate and safe when compared to Pulse Contour Cardiac Output (PiCCO) and RHC, the current gold standard in continuous hemodynamic monitoring [11,12,13,14,15]. Gellert et al. analyzed ten previous studies that compared invasive and non-invasive CO measurements and found similar standard deviations (SD) and percentage errors (PE), as well as generally lower non-invasive measurements, consistent with our findings (mean SD: 0.4, mean PE: 43%) [11]. Overall, our findings in patients with severe AS were consistent to prior studies in different clinical settings.

Third, all evaluated parameters showed significant discrepancies between invasive and non-invasive measurements; however, none of the analyzed parameters fell below the threshold of acceptable bias of 20% and percentage error of 30%, as previously used by Di Cori et al. [19].

Fourth, continuous measurements reveal a drop in BP during valve deployment, as well as a spike in CO, CI and SV. This might be explained by a temporary obstruction of the LVOT during valve deployment and a subsequent increase in output after the obstruction is released. There is limited data on the effect of valve deployment on hemodynamic parameters. However, Alperi et al. have shown a significant drop in valvular gradients periprocedurally, which fits our findings [23].

Fifth, our study did not allow for an evaluation of the impact on clinical endpoints due to a lack of events. This is unlikely due to an unusually healthy patient cohort, as our baseline characteristics were comparable to those in other studies [24,25]. Despite representing one of the largest populations investigating the peri-interventional application of non-invasive monitoring in patients undergoing TAVI, our findings suggest that a larger sample size and longer follow-up period may be necessary to validate these observations further. The absence of clinical events highlights the need for extended monitoring to capture more comprehensive data on potential clinical outcomes. Future studies should investigate possible benefits of ClearSight^®^ in earlier diagnosis of peri- and immediately post-TAVI complications.

ClearSight^®^ has shown a level concordance of 84–100% with PAC and PiCCO, indicating that it generally follows the same trend as these gold-standard measurements, though the absolute values may differ [16]. This discrepancy might be due to inherent differences in measurement techniques, with invasive methods providing more direct and possibly more precise assessments of hemodynamic parameters. Additionally, ClearSight^®^ has shown good correlation with other methods such as FloTrac and echocardiography, but large differences in absolute values have been noted [12,26].

## 5. Conclusions

In conclusion, while non-invasive measurements offer a valuable alternative for continuous hemodynamic monitoring and can effectively indicate general trends observed with invasive techniques, they currently lack the precision and accuracy of invasive methods. The discrepancies in BP, CO, CI, SV measurements between the two modalities highlight the inherent limitations of non-invasive techniques. Despite these limitations, non-invasive systems like ClearSight^®^ have demonstrated concordance with invasive methods and provide significant clinical benefits, such as earlier detection of hypotensive episodes and a lower risk of complications. However, for critical care settings where precise hemodynamic monitoring is essential, invasive techniques remain the gold standard. Further research with larger sample sizes and extended follow-up periods is warranted to enhance the accuracy and reliability of non-invasive monitoring systems and to fully evaluate their impact on clinical outcomes.

## Figures and Tables

**Table 1:** Characteristics of patients.

Variable	N	N = 50 ^1^
Basic characteristics		
Age	50	82.0 (78.0, 85.8)
Sex	50	
Male		25 (50%)
Female		25 (50%)
BMI	49	26.0 (23.6, 31.0)
Preconditions		
Diabetes	50	17 (34%)
Dyslipidemia	50	33 (66%)
Hypertension	50	41 (82%)
Euro SCORE II	50	1.90 (1.40, 4.30)
STS Calculated risk of mortality	50	2.40 (1.65, 4.02)
eGFR (Cockroft-Gault)	50	70 (54, 79)
Hemoglobin	50	131 (116, 141)
LVEF	45	60 (55, 65)
Atrial fibrillation or flutter	49	9 (18%)
Peripheral artery disease	50	2 (4.0%)
Coronary artery disease	50	18 (36%)
NYHA-Score	48	
1		1 (2.1%)
2		29 (60%)
3		17 (35%)
4		1 (2.1%)
Previous intereventions		
Myocardial infarction	50	4 (8.0%)
Percutatious coronary intervention	50	5 (10%)
Coronary artery bypass surgery	50	2 (4.0%)
Aortic valve surgery	50	0 (0%)
TAVI	50	0 (0%)
Medication		
Aspirin	50	27 (54%)
Clopidogrel	50	6 (12%)
NOAC	50	5 (10%)
Warfarin/Marcoumar	50	1 (2.0%)
ACEi/ARB	50	28 (56%)
Calcium channel blocker	50	9 (18%)
Statin	50	32 (64%)
Beta blocker	50	23 (46%)
Diuretics	50	24 (48%)

^1^ Median (IQR); n (%); NOAC = Novel Oral Anticoagulants; ACEi = ACE inhibitor; ARB = Angiotensin receptor blocker.

**Table 2:** Summary statistics of the BA analyses comparing non-invasive ones (ClearSight^®^) measurements (aortal for SV, CO, and CI and radial for BP) and invasive ones.

Variable	Absolute Bias	SD	LoA	CE	PE [%]
Stroke Volume [mL]	−11.08	27.43	102.48	0.39	77.21
Systolic Blood Pressure [mmHg]	−20.20	27.15	102.76	0.22	43.71
Diastolic Blood Pressure [mmHg]	−5.6	15.61	59.00	0.27	54.49
Cardiac Output [L/min]	−0.70	1.25	4.66	0.28	56.12
Cardiac Index [L/min/m^2^]	−0.44	0.71	2.66	0.29	58.34
Radial Systolic Blood Pressure [mmHg]	−17.92	22.07	86.33	0.19	37.78
Radial Diastolic Blood Pressure [mmHg]	−8.62	10.41	40.70	0.20	39.24

BA = Bland–Altman, SD = standard deviation, LoA = limit of agreement, CE = coefficients of error, PE = percentage error.

## Data Availability

The data that support the findings of this study are available from the corresponding author, TN, upon reasonable request.

## Abbreviations

The following abbreviations are used in this manuscript:

AS	Aortic valve stenosis
TAVI	Transcatheter Aortic Valve Implantation
SAVR	Surgical Aortic Valve Replacement
RHC	Right heart catheterization
CO	Cardiac output
CI	Cardiac index
SV	Stroke volume
SVI	Stroke volume index
SVV	Stroke volume variation
SVR	Systemic vascular resistance
PiCCO	Pulse Contour Cardiac Output
PAC	Pulmonary artery catheterization
BP	Blood pressure
HR	Heart rate
IQR	Interquartile range
SD	Standard deviation
LoA	Limits of agreement
CE	Coefficients of error
PE	Percentages of error
LVEF	Left ventricular ejection fraction
NYHA	New York Heart Association
VARC3	Valve Academic Research Consortium3
EuroSCORE II	European System for Cardiac Operative Risk Evaluation II
STS	Society of Thoracic Surgeons
